# Somatic Symptoms Evoked by Exam Stress in University Students: The Role of Alexithymia, Neuroticism, Anxiety and Depression

**DOI:** 10.1371/journal.pone.0084911

**Published:** 2013-12-18

**Authors:** Matthias Zunhammer, Hanna Eberle, Peter Eichhammer, Volker Busch

**Affiliations:** 1 Department of Experimental Psychology, University of Regensburg, Regensburg, Germany; 2 Department of Psychiatry, University of Regensburg, Regensburg, Germany; Leiden University Medical Centre, Netherlands

## Abstract

**Objective:**

The etiology of somatization is incompletely understood, but could be elucidated by models of psychosocial stress. Academic exam stress has effectively been applied as a naturalistic stress model, however its effect on somatization symptoms according to ICD-10 and DSM-IV criteria has not been reported so far. Baseline associations between somatization and personality traits, such as alexithymia, have been studied exhaustively. Nevertheless, it is largely unknown if personality traits have an explanatory value for stress induced somatization.

**Methods:**

This longitudinal, quasi-experimental study assessed the effects of university exams on somatization — and the reversal of effects after an exam-free period. Repeated-observations were obtained within 150 students, measuring symptom intensity before, during and after an exam period, according to the Screening for Somatoform Symptoms 7-day (SOMS-7d). Additionally, self-reports on health status were used to differentiate between medically explained and medically unexplained symptoms. Alexithymia, neuroticism, trait-anxiety and baseline depression were surveyed using the Toronto-Alexithymia Scale (TAS-20), the Big-Five Personality Interview (NEO-FFI), the State Trait Anxiety Inventory (STAI) and Beck’s Depression Inventory (BDI-II). These traits were competitively tested for their ability to explain somatization increases under exam stress.

**Results:**

Somatization significantly increased across a wide range of symptoms under exam stress, while health reports pointed towards a reduction in acute infections and injuries. Neuroticism, alexithymia, trait anxiety and depression explained variance in somatization at baseline, but only neuroticism was associated with symptom increases under exam stress.

**Conclusion:**

Exam stress is an effective psychosocial stress model inducing somatization. A comprehensive quantitative description of bodily symptoms under exam stress is supplied. The results do not support the stress-alexithymia hypothesis, but favor neuroticism as a personality trait of importance for somatization.

## Introduction

Somatization has been defined as the “tendency to experience and communicate somatic distress in response to psychosocial stress and to seek medical help for it” [1]. Although the re-definition of somatization as a clinical concept and its classification under the psychiatric category “somatic symptom disorders” is a matter of ongoing debate [2] there is consensus that medically unexplained symptoms (MUS) and a stress-related etiology belong to its core features.

### Somatization and personality traits – Is alexithymia the key concept?

The causes of somatization have been hypothesized to be multifactorial, involving several mechanisms (for review see: [1,3,4]). Evidence suggests co-occurrence and shared mechanisms with negative affect, anxiety [5], neuroticism [6,7] and alexithymia [8]. Especially alexithymia, the inability to identify, describe and differentiate emotions, has attracted considerable attention as a potential predisposing factor for somatization (for review see: [9]). However, the belief that alexithymia causes or contributes to somatization is mainly based on cross-sectional studies, which do not allow causal inferences [8,9]. Several authors therefore underlined the need for more longitudinal studies [8–10]. Mechanistically, alexithymia has been hypothesized to affect somatization by modulating physiological responses to stress [11]. Although this “stress-alexithymia hypothesis” has been experimentally tested on measures of autonomic reactivity [12,13], its relevance for somatization induced by a naturalistic psychosocial stressor has, to our knowledge, not been tested to date.

### The effects of exam stress on somatization are unknown

The effectiveness of exam stress as a model of psychosocial stress has repeatedly been shown on immunological [14–16], neuroendocrine [16,17], physiological and psychological [18–21] parameters. Despite these associations, exam stress has not been used to investigate predisposing factors of somatization so far. To our knowledge only Koh and colleagues (2006) [21] determined the effects of exam stress on somatization, showing a significant positive relationship in 38 participants. Still, no quantitative description of somatization symptoms under exam stress is available, although the somatic symptoms of acute exam anxiety have been assessed systematically [22,23].

The present study investigated somatization by exploring increases in MUS as a reaction to naturalistic psychosocial stress and by competitively testing the explanatory value of several personality traits including alexithymia.

Our first aim was to provide a quantitative description of somatic symptom increases under exam stress including all 53 physical symptoms from the somatization symptom lists of ICD-10 and DSM-IV. It was hypothesized that an exam period would affect total symptom scores, as well as distinct symptoms. Both were expected to increase under exam stress and return to baseline after a period without exams.

Our second aim was to test the predictive value of alexithymia and such related concepts as neuroticism, trait anxiety and depression for *increases* in somatization under exam stress. It was hypothesized that alexithymia would correlate positively with somatic symptom increases during exam stress, according to the stress-alexithymia hypothesis, and show a stronger association with these increases than neuroticism, state anxiety, or depression.

## Methods

The study was approved by the ethics committee of the University of Regensburg and conforms to the Declaration of Helsinki. Informed consent was obtained. Data were collected and analyzed pseudonymously. Written informed consents were obtained.

### Study design

**Figure 1:**
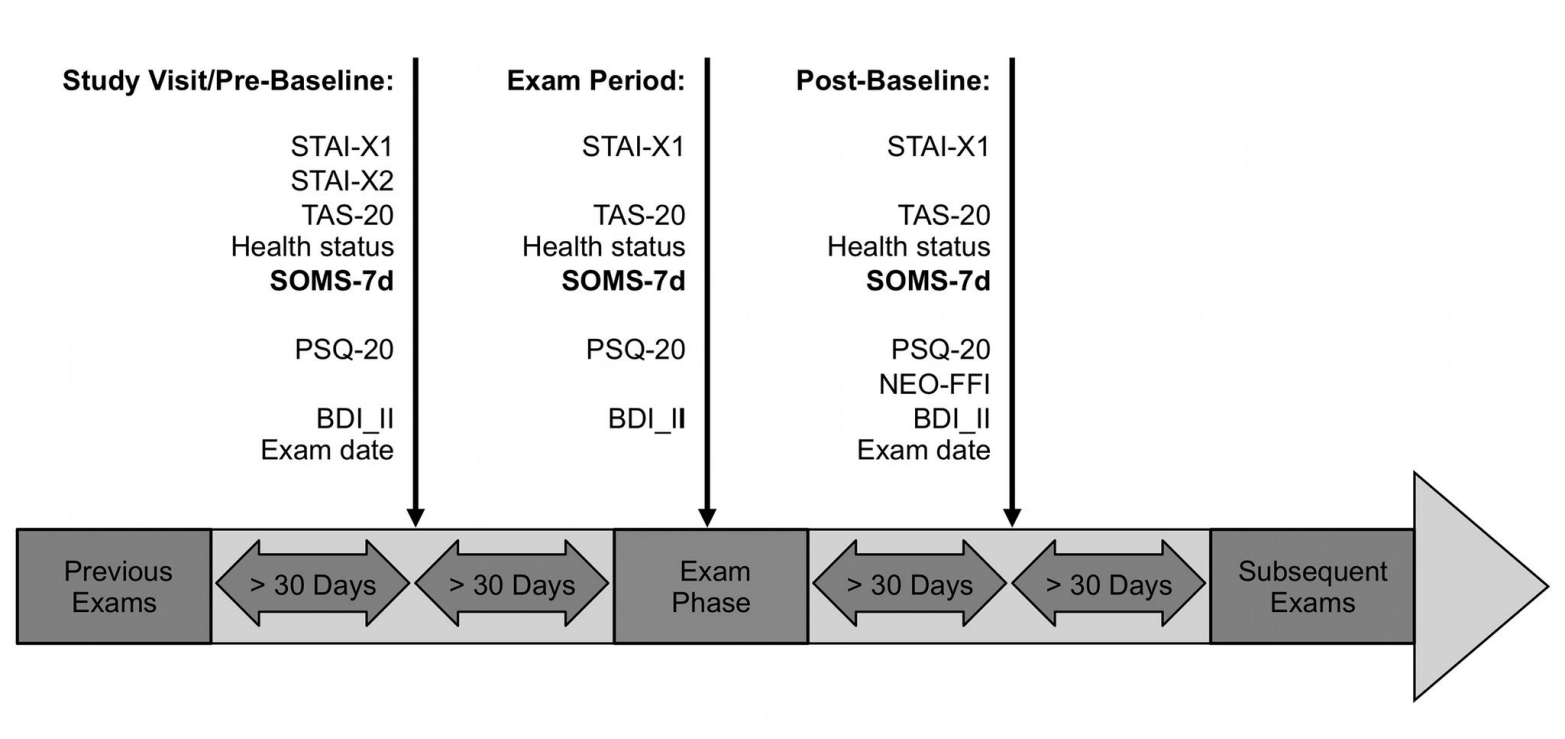
Timeline.

We conducted a longitudinal, quasi-experimental study with a natural event, a reversal period and several control variables. The natural, predictive event was defined as a major university exam. Since exam types vary between academic disciplines, this was specified as an exam being prerequisite for graduation, or contributing to the university degree. Since exams are often clustered, the most fearsome and/or distressing exam according to participants’ choice was selected. The intervention reversal period was defined as a subsequent exam-free period of 30 days. Repeated observations were obtained at three times within participants: before (pre-baseline) and immediately after the predictive exam (exam period), as well as after reversal period (post-baseline). To guarantee that exam stress did not affect baselines, data was only included when participants reported no exam within the last and next 30 days. Moreover, to assure that the effect of the exam was maximal, data for exam period was excluded when participants failed to submit their survey within 3 days. Figure 1 provides an overview of the study’s time course.

To increase internal validity, additional control measures were recorded: acute infections, injuries or exacerbations of pre-existing conditions, i.e. medically explained symptoms (MES), pose an obvious alternative explanation for increases in symptom reports. Therefore, descriptive participant reports of current health status were surveyed, categorized and subsequently tested for effects of exam period. Additional measures of stress, state-anxiety and negative mood were taken to reassure that exam period was an effective psychosocial stressor.

### Participants

For the present study 150 students of the University of Regensburg and the Regensburg University of Applied Sciences were recruited systematically across all faculties by advertisement via bulletins, flyers and personal appeal at academic lectures. Investigators’ relatives, friends and colleagues were excluded from participation. Past or present internal, neurological, hormonal or psychiatric disorders were evaluated in a structured interview at study inclusion. Participants with acute conditions and in medical treatment were excluded from participation. Individuals with past or chronic disorders in stable remission were included, but their status was addressed as a potential confound in analysis. Participants received a compensation of 8 Euros per hour.

### Procedure

The only study visit was scheduled at least 30 days before the first major exam. Written informed consent, medical history and exam dates were obtained. If necessary, exam dates were followed up by telephone interview and participants were advised to report any exams that had to be re-scheduled. In addition, exam dates were retrieved at the end of the post-baseline session. All questionnaires were obtained using online forms. Each participant received an e-mail containing a web-link to an online platform and instructions on the day of study visit one (pre-baseline), the day of the selected exam (exam period) and one month after the last exam according to the participant’s specifications (post-baseline) (see Figure 1). All online-questionnaires were identical to the paper versions, with the exception that missing items were prohibited by forced-choice settings.

### First Aim – State Questionnaires

Symptom intensities of 53 physical symptoms from the somatization symptom lists of ICD-10 and DSM-IV were measured according to the Screening for Somatoform Symptoms 7-day version (SOMS-7d) [24,25], with the difference that instructions asked participants to report perceived impairment for all symptoms, *without* requesting participants to differentiate between MUS and MES. The SOMS-7d was designed to measures impairment by 53 typical somatization symptoms, such as “headache”, “bloating” or “back ache” on a 5-point Likert-scale within the last seven days: Scores are 0 (symptom absent or not impairing), 1 (mild), 2 (medium), 3 (severe) and 4 (very severe). SOMS-7d “symptom index” was the main outcome measure, calculated by summation of all items [24–26].

Four custom items surveying current health status within the last seven days were used to differentiate between MES and MUS: Item one asked if the participant was feeling healthy today. Item two asked if medical treatment or counsel had recently been taken. Item three asked for the occurrence of any disease or injury and item four for exacerbations of pre-existing conditions. If any item was answered with “yes”, participants were required to enter a detailed description of their condition and symptoms into an open form field. Based on these items and responses, three of the authors (M.Z., H.E., V.B.) independently categorized sessions as “evidence for MES” (yesMES), or “no evidence for MES” (noMES). Where raters’ categorization did not match, sessions were classified as yesMES. This approach to differentiate between MES and MUS was chosen to preclude that the causal attribution of participants could bias somatization scores.

The Perceived Stress Questionnaire (PSQ-20) [27] was used to confirm that the exam period was perceived as stressful. The PSQ-20 is a shortened, German adaptation of the original PSQ-30 [28], with good reliability (Cronbach’s alpha >.80). Perceived stress is measured using 20 negatively and positively worded items, such as “Your problems seem to be piling up”, “You have trouble relaxing”, or “You have enough time for yourself”. Items are rated on a 4-point likert scale (1 “almost never”, 2 “somtimes”, 3 “often”, 4 “usually”) [27].

The German versions of the State-Trait Anxiety Inventory – State Form (STAI-G-X1) [29] and Beck’s Depression Inventory II (BDI-II)[30], were used to survey state anxiety and depression levels at all time-points as an intervention check. For consistency, the BDI-II was adapted to a time-span of seven days.

### Second Aim – Trait Questionnaires

Alexithymia and three competing explanatory personality traits were examined for their explanatory value for SOMS-7d scores: The 20-item version of the Toronto-Alexithymia Scale (TAS-20) [31–33] was the primary measure of alexithymia with respect to our second aim. The TAS-20 surveys alexithymia on the three dimensions “Difficulty identifying feelings”, “Difficulty describing feelings” and “Externally oriented thinking style”. It uses negatively and positively worded items like: “I’m often confused about what emotion I am feeling”, “I am able to describe my feelings easily” and “Being in touch with emotions is essential”. Items are rated on a five-point Likert scale ranging from 1 (strongly disagree) to 5 (strongly agree). From the perspective of test- and measurement theory the 20-item version of the TAS-20 is the most robust and time-economic instrument to measure Alexithymia currently available. However it has been criticized for its lack of discriminative validity and the fact that it requires a self-evaluation — the very feature alexithymia individuals are impaired in by definition [34–36]. Although defined as a trait measure, we collected the TAS-20 at all three time-points, to follow up reports on its doubtful temporal stability as a side-aim [37]. For all other analyses the post-baseline score of the TAS-20 was used.

Trait-anxiety was surveyed using the State-Trait Anxiety Inventory – Trait Form (STAI-G-X2) [29]. Trait neuroticism was measured by using the Big Five Personality Inventory NEO-FFI [38]. Trait depression was defined as the mean BDI-II score of both baselines. Although the BDI-II is mostly used as a measure of state depression it has been reported to accurately assess trait-like characteristics [39].

### Statistics

Statistics were processed with SPSS 21.0.0.0 for Mac OS (Statistical Product and Service Solutions Inc., Chicago, IL, USA), graphs were created using GraphPad Prism 5.0 (GraphPad Software, Inc, La Jolla, CA, USA). Statistical tests were performed at a two-tailed α<.05. Means are given ±standard deviation if not denoted otherwise.

For aim one, a mixed linear model was used to estimate the effect of fixed factor TIME with the levels pre-baseline, exam and post-baseline on SOMS-7d symptom index, using SPSS’s GENLINMIXED function. To control for potential confounds, fixed factors MES with the levels yesMES and noMES, as well as factor DISORDER with levels yesDIS (i.e. stable past or chronic disorders) and noDIS (i.e. no disorders reported) were included into the model. To account for individual differences in SOMS-7d intensity score, a random intercept was added for each participant. An autoregressive covariance matrix (AR1) was used to model repeated covariance within sessions. Robust estimation options and Satterthwaite-approximation were used to account for potential violations of model assumptions and correction of the estimated degrees of freedom for unequal sample sizes. The SPSS’s syntax for this basic model was added to Supplement S1. The mixed model allows analyzing repeated-measures data without list-wise exclusion of missing values. Therefore all sessions meeting the deadlines in respect to past/upcoming exams were included in the analysis, even when a participant missed one or two sessions or deadlines.

All other control variables besides MES were tested for effects of TIME analog to the linear mixed model for somatization scores.

For MES, a chi-square test was used to determine if the proportion of reported infections/injuries during exam period differed from baselines.

Further the mixed model described above was applied to address the question if the TAS-20 is a stable trait measure over TIME. In addition inter- (Pearson’s correlation coefficient) and intra-class correlations (ICC) for the TAS-20 were computed.

Friedman’s tests were used for an item-by-item analysis of the SOMS-7d in order to identify symptoms increasing during exam period. For this item analysis an adapted α-Level of .001 was used to control for multiple-comparisons, and post-hoc Wilcoxon’s paired rank tests were performed to confirm increases between exam period and at least one baseline.

Aim two was to identify the best explanatory trait variable for somatic symptoms at baseline and symptom increase under exam stress. Traits were alexithymia (TAS-20), neuroticism (NEO-FFI), trait anxiety (STAI-X-2) and trait depression (BDI-II).

First, a correlation table using Kendall’s tau-b was created to explore monotonous relationships. Kendall’s tau-b is the non-parametric correlation coefficient of choice for symptom rating scales, especially when comparisons are made between (sub-) samples of different size [40]. For correlational analysis, symptom index at baseline was defined as the mean of both baseline sessions and somatization increase was defined as symptom index during the exam period minus baseline.

Correlation analysis was followed by a modeling approach to determine the best trait predictor of somatization symptoms. Eight variants of the basic model described above (TIME, MES and DISORDER (df_1_=4)) were compared: Four models were created by adding one of the trait variates ALEXITHYMIA, NEUROTICISM, ANXIETY or DEPRESSION (df_1_=5). Four further models included the respective first-degree trait-by-TIME interaction (df_1_=7) in addition. An individual random slope parameter for each trait variate was added to each model to keep the random effects structure of the model “maximal” [41]. The model syntax for this analysis is given in Supplement S1. Finally, Akaike’s Information Criterion for finite sample sizes (AICc) and Akaike Weights [42] were used to determine the best-fitting model.

## Results

### Sample description

**Figure 2:**
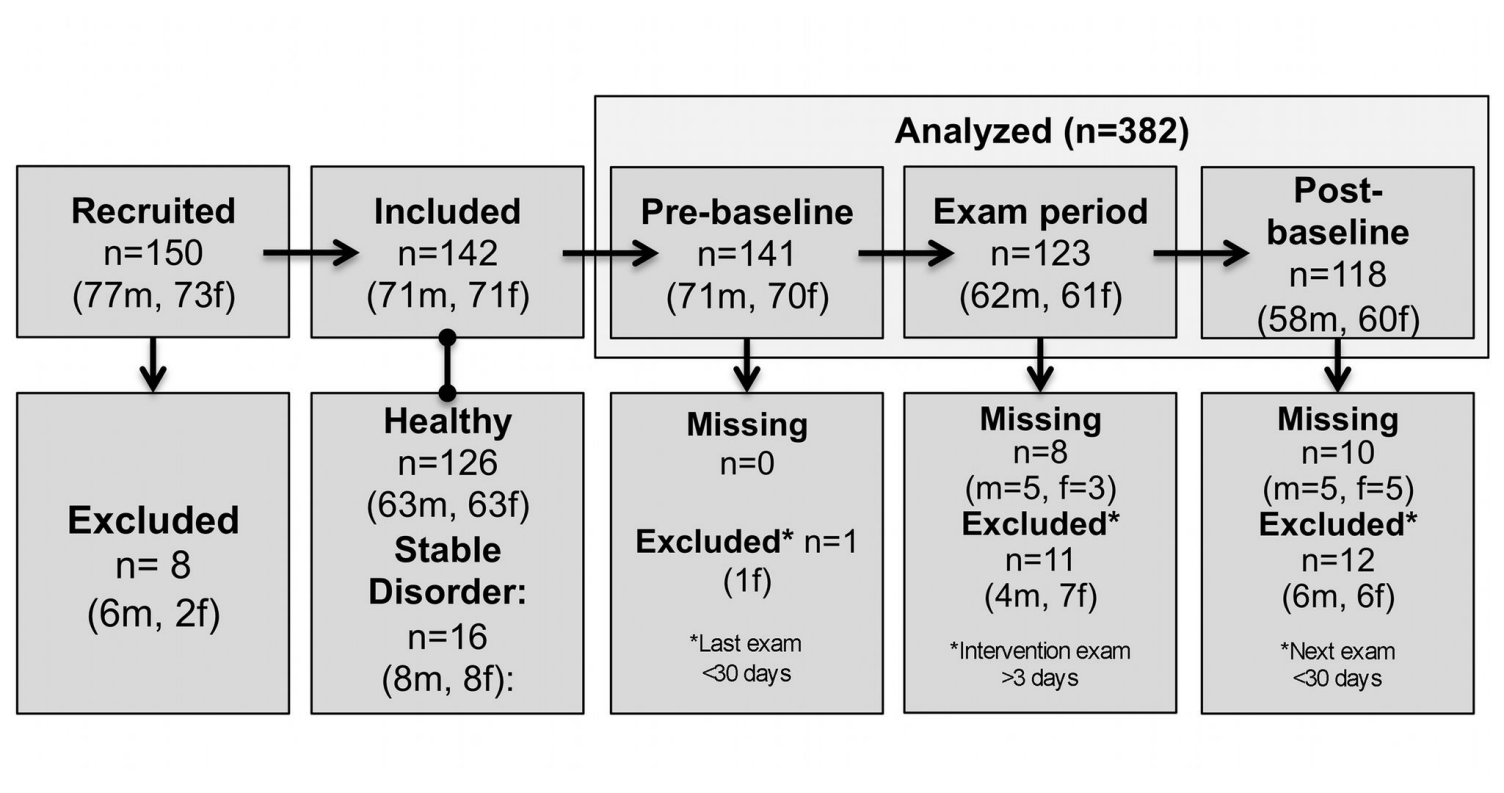
Participant flow-chart.

Figure 2 gives an overview of participant flow. Analysis was based on 142 participants (71 male, 71 female), of which 107 contributed three, 26 two and 9 one valid session(s). Mean age at study inclusion was 22.2±2.5 (range: 18-33) years. Participants with past or chronic internal, neurological and psychiatric disorders in stable remission constituted 11% (8 male, 8 female) of the sample. The mean number of exams reported was 4.9±2.1, ranging from 1 to 12. At mean, the exam survey was submitted 1.0±1.6 days after the exam defined as the intervention. For additional sample information see Supplement S1.

### Effects of Exam Stress on Somatization

In summary, the symptom index and all intervention check variables assessing stress, depression and state anxiety were found to significantly increase during the exam period compared to both pre- and post-exam baselines. Inclusion of the factors MES and DISORDER significantly improved model fit for the prediction of symptom index (Δdf_1_=+2, ΔAICc=-20.29) and most control variables. Descriptive results are shown in Table 1, the corresponding linear mixed model results are shown in Table 2.

**Table 1:** The effects of exam period on measures of somatic symptoms and stress: Descriptive results.

	**Pre-baseline**	**Exam period**	**Post-baseline**
	n=141	n=123	n=118
	Mean±SD		
**Primary variable:**			
**SOMS-7d**	11.1±8.9	18.2±14.5	9.7±9.0
**Control variables:**			
**PSQ-20**	33.6±17.5	54.0±19.3	29.8±18.3
**BDI-II**	6.6±6.4	11.5±7.0	5.2±5.4
**STAI-G-X1 state**	36.9±8.1	42.74±12.3	37.1±10.3
**Side aim: Temporal stability of the TAS-20**			
**TAS-20**	45.3±9.7	45.9±10.1	44.6±10.2

Abbreviations: BDI-II: Beck’s Depression Inventory; PSQ-20: Perceived Stress Questionnaire; SOMS-7d: Screening for Somatoform Symptoms 7-day version; STAI-G-X1 state: State-Trait Anxiety Inventory – State Form; TAS-20: Toronto Alexithymia Scale 20-item version.

**Table 2:** The effects of exam period on measures of somatic symptoms and stress: Mixed model results.

	**Model**	**Coefficient estimates**
	df_1_=4	β±SEM
**Primary variable:**		
**SOMS-7d**	df_2_=6, F=18.92, **p<.001**	β_Exam_=8.89±1.06, t=8.42, **p=.009**
		β_preBL_=1.77±0.69, t=2.553, **p=.012**
		β_MESyes_=2.44±1.11, t=2.20, **p=.032**
		β_yesDIS_=7.63±3.15, t=2.42, p=.076
**Control variables:**		
**PSQ-20**	df_2_=168, F=55.68, **p<.001**	β_Exam_=24.22±1.95, t=12.45, **p<.001**
		β_preBL_=3.98±1.65, t=2.410, **p=.017**
		β_yesMES_=0.27±2.12, t=0.13, p=.899
		β_yesDIS_=15.20±4.25, t=3.58 **p=.001**
**BDI-II**	df_2_=63, F=37.02, **p<.001**	β_Exam_=6.60±0.61, t=10.80, **p<.001**
		β_preBL_=1.69±0.50, t=3.17, **p=.003**
		β_yesMES_=1.04±0.67, t=1.55, p=.124
		β_yesDIS_ =4.49±1.54, t=2.92, **p=.005**
**STAI-G-X1 state**	df_2_=68, F=10.34, **p<.001**	β_Exam_=5.86±1.19, t=4.92, **p<.001**
		β_preBL_=0.79±0.90, t=0.88, p=.930
		β_yesMES_=0.69±1.20, t=0.58, p=.566
		β_yesDIS_=6.32±2.40, t=2.63, **p=.011**
**Side aim: Temporal stability of the TAS-20**		
**TAS-20**	df_2_=377, F=2.71, **p=.030**	β_Exam_=1.80±0.60, t=3.00, **p<.003**
		β_preBL_=0.79±0.56, t=1.41, p=.164
		β_yesMES_=0.46±0.93, t=0.49, p=.623
		β_yesDIS_=4.14±3.18, t=1.30, p=.359

All linear mixed models included factors TIME (pre-baseline, exam, post-baseline), MES (yesMES, noMES) and DISORDER (yesDIS, noDIS) and assumed a normal distribution. Degrees of freedom (df_2_) may vary due to inequal sample sizes and the Satterthwaite approximation used. All analyses were based on 142 subjects contributing 382 data-points. Abbreviations: BDI-II: Beck’s Depression Inventory; GLM: Generalized Linear Model; MES: Medically Explained Symptoms; preBL: pre-baseline; PSQ-20: Perceived Stress Questionnaire; SOMS-7d: Screening for Somatoform Symptoms 7-day version; STAI-G-X1: State-Trait Anxiety Inventory – State Form; TAS-20: Toronto Alexithymia Scale 20-item version; yesDIS: With stable past/chronic disorders; yesMES: Evidence for MES.

A chi-square test indicated that the proportion of participants reporting acute infections and/or injuries (evidence for MES) during exam period (8.1%) was significantly lower (df=2, χ^2^=7.45, p=.024) than at pre- (19.1%) and (18.6%) and post- exam baseline.

Almost all participants (95.0%) reported at least one symptom causing mild impairment according to the combined ICD-10 and DSM-IV list at baseline. The cumulative proportions of participants reporting at least one moderate, severe, or very severe symptom were 64.9%, 28.6% and 2.7% respectively. These proportions increased under exam stress (mild: 97.6%, medium: 80.5%, severe: 51.2%, very severe: 16.3%). The eleven SOMS-7d items listed in Table 3 were found to be significantly elevated under exam stress according to Friedman’s-tests and post-hoc tests at α≤.001. A full description of results for all SOMS-7d –items is available online (see: Table S1). 

**Table 3:** Results for somatization items significantly increasing under exam stress.

**Item**	**Symptom**	**Pre-baseline**	**Exam period**	**Post-baseline**	**Relative increase in prevalence**		**Exam effect**
					**any**	**severe**	
		n=141	n=123	n=117			df=2
		Mean±SD			% of baseline		Χ^2^_F_, sig
**1**	**Headache**	0.82±0.92	1.33±1.11	0.71±0.78	30.7	258.8	26.70, ****
**2**	**Abdominal pain**	0.65±0.89	0.93±0.98	0.64±0.79	30.7	123.1	14.91, ***
**3**	**Back pain**	0.71±0.86	1.24±1.12	0.75±0.84	29.0	474.3	26.85, ****
**4**	**Joint pain**	0.33±0.69	0.59±0.88	0.34±0.64	53.2	76.5	14.17, ***
**10**	**Nausea**	0.45±0.71	0.76±0.90	0.41±0.66	53.8	317.8	23.64, ****
**12**	**Discomfort/churning around stomach**	0.72±0.94	1.24±1.10	0.54±0.83	79.8	199.5	43.44, ****
**17**	**Loss of appetite**	0.30±0.68	0.67±0.97	0.22±0.59	136.2	222.9	26.36, ****
**20**	**Frequent diarrhea**	0.22±0.60	0.50±0.91	0.19±0.53	105.3	227.9	14.24, ***
**30**	**Excessive tiredness after mild exertion**	0.50±0.85	1.03±1.21	0.37±0.62	66.7	487.5	29.88, ****
**32**	**Sexual indifference**	0.35±0.69	0.57±0.83	0.23±0.55	80.9	164.8	16.08, ***

Symptoms were surveyed according to the Screening for Somatoform Symptoms 7-day version (SOMS-7d). Effects of exam period were tested using Friedman’s test (Χ^2^_F_). The alpha-Level was set to p≤.001 to correct for multiple comparisons. Significant results are depicted as **** p≤.0001, *** p≤.001. All post-hoc differences between baselines and exam period were significant as tested with Wilcoxon’s paired rank tests (results not shown). Increases in symptom prevalence are shown in % of valid cases for any severity (score≥1) and severe/very severe only (score≥3) at baseline.

### The explanatory value of personality traits on stress induced somatization

Mean TAS-20 baseline score was 44.9±9.5 and therefore roughly 4 units below norm values for the German population [33]. Accordingly, only 8.9%, or 19.3%, of participants reached a TAS-20 score of 61 or 52.5 — cut-off values suggested for the diagnosis of “alexithymic” [33]. The significant differences in mean response found for TAS-20 sum scores over time (see Table 2) were followed up by computing Pearson’s coefficient and the intra-class correlation coefficient (ICC): Correlations were r=.788 for Survey 1 and 2, r=.804 for Survey 2 and 3 and r=.787 (all p<.001) for Survey 1 and 3. Single ICC was .786, mean ICC .917.

NEO-FFI-neuroticism scores [38] were close to normal values with a mean of 21.0±7.2 points. Mean BDI-II score was 6.0±5.2 and therefore 4 units below a normal student sample [43]. STAI-G-X2 trait anxiety scores were at 39.2±8.7 points and therefore 4 units above a normal student sample [29].

Correlations between somatic symptoms and trait variables according to Kendall’s τ-b are shown in Table 4. NEO-FFI-neuroticism was the only significant trait that correlated with somatic symptom increase during exam period, showing a positive monotonous relationship. All trait variables correlated with symptom index at baseline. All trait variables were considerably inter-correlated.

**Table 4:** Correlations between somatic symptoms at baseline, somatic symptom increase during an exam period and personality traits.

	**SOMS-7d baseline**	**SOMS-7d increase**	**TAS-20 alexithymia**	**NEO-FFI neuroticism**	**STAI-G-X2 trait anxiety**
**SOMS-7d increase**	n=134, τ=.074, p=.212				
**TAS**-**20 alexithymia**	**n=132, τ=.228, p<.001**	n=131, τ=-.050, p=.412			
**NEO-FFI neuroticism**	**n=132, τ=.359, p<.001**	**n=131, τ=.152, p=.012**	**n=132, τ=.270, p<.001**		
**STAI-G-X2 trait -anxiety**	**n=142, τ=.295, p<.001**	n=134, τ=.074, p=.212	**n=132, τ=.319, p<.001**	**n=132, τ=.514, p<.001**	
**BDI-II baseline depression**	**n=142, τ=.413, p<.001**	n=134, τ=.066, p=.271	**n=132, τ=.327, p<.001**	**n=132, τ=.470, p<.001**	**n=142, τ=.482, p<.001**

All tests were performed using Kendall’s τ-b. Abbreviations: BDI-II: Beck’s Depression Inventory; NEO-FFI: Big Five Personality Inventory, Neuroticism Subscale; SOMS-7d: Screening for Somatoform Symptoms 7 day version; STAI-G-X2: State-Trait Anxiety Inventory – Trait Form; TAS-20: Toronto Alexithymia Scale, 20-item version.

An information theory driven model selection approach was applied to determine the best explanatory trait variable for the obtained somatization scores. AICc weights indicated that the basic model plus NEUROTICISM and NEUROTICISM*TIME (df_1_=7) was the best-fitting model, with a probability of 0.97 relative to all equal or smaller models according to Akaike weights. The second-best model was the basic model plus the main effect of NEUROTICISM only, with a probability of only .03. The competing models with TAS-20 and TAS-20*TIME (ΔAICc=+31.59), STAI-G-X2 and STAI-G-X2*TIME (ΔAICc=+105.22) and BDI-II and BDI-II*TIME (ΔAICc=+81.93) had only marginal likelihoods of being the best-fitting model.

**Figure 3:**
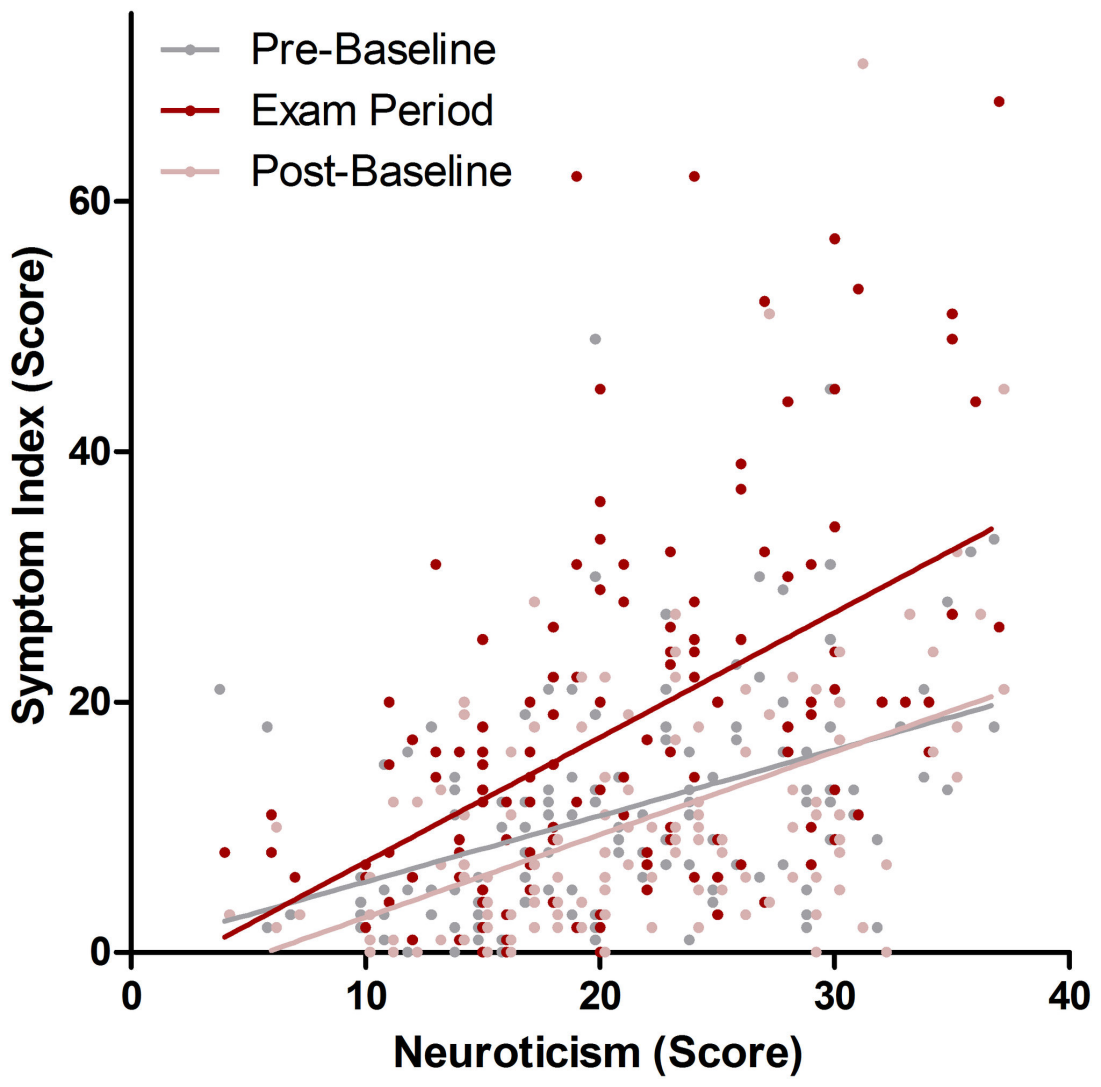
The relationship between trait neuroticism, somatic symptoms at baseline and under exam stress. There was a significant interaction between NEUROTICISM and exam period (β=0.42±0.13(SEM), t=3.30, p=.020), even when accounting for medically explained symptoms and pre-existing disorders. Raw data are shown and simple linear interpolation lines were added for illustrative purposes. To reduce overlap, data points for pre- and post- baseline were shifted by -0.5 and +0.5 points along the x-axis, respectively,

The final model (F=14.63, df_1_=7, df_2_=6, p=.002) showed a significant main effect of NEUROTICISM (β=0.44±0.10(SEM), t=4.34, p<.001) and MES (β=2.55±1.20(SEM), t=2.10, p=.039), as well as a positive interaction between NEUROTICISM and TIME (F=5.54, df_1_=2, df_2_=10, p=.025), driven by a significant interaction of coefficients NEUROTICISM*exam period (β=0.42±0.13(SEM), t=3.30, p=.020). The factor DISORDER fell short of the criterion of significance (F=2.58, df=16, p=.128), which was also missed by all other coefficients. A graphical display of the relationship between symptom index, NEUROTICISM and TIME is given in Figure 3.

The models including TAS-20 (F=16.39, df_1_=7, df_2_=3, p<.017), STAI-G-X2 (F=15.01, df_1_=7, df_2_=7, p<.001) and BDI-II (F=16.76, df_1_=7, df_2_=8, p<.001) were all explaining a significant amount of variance, however the coefficients TAS-20*exam period (β=0.101±0.12(SEM), t=0.86, p=.553), STAI-G-X2*exam period (β=0.31±0.13(SEM), t=2.46, p=.147) and BDI-II*exam period (β=0.41±0.23(SEM), t=1.78, p=.183) failed to do so.

Original data for the present results have been made publicly available as a download. However, the following changes to the raw data file were made: Birthdate, exam date, date of study inclusion, information on illicit drug use, subject of study, original description of medical history and original description of current illness/injuries were deleted and/or replaced by summary variables to ensure participant’s privacy.

## Discussion

This quasi-experimental study was conducted to provide a comprehensive quantification of somatic symptoms under exam stress in healthy students and to evaluate whether personality traits like alexithymia can explain their occurrence.

### Exam stress increases somatization — typical symptoms

During an exam period symptom scores showed highly significant increases compared to pre- and post-baselines, even when accounting for participant reports of infections/injuries and pre-existing disorders statistically. This was paralleled by increases in perceived stress, depression and anxiety. These findings confirm that academic exam periods represent an effective model for psychosocial stress, with a significant impact on somatization. Bodily complaints increased across several domains encompassing pain, gastro-intestinal and autonomic symptoms. Significant increases during the exam period were mainly found for symptoms with a high prevalence at baseline, e.g: headache, back pain, abdominal pain, and nausea. An exception of this trend was bloating, which did not significantly increase although being one of the most common symptoms. The symptom with the highest absolute increase in prevalence across all severities was discomfort/churning around the stomach, which is synonymous to the proverbial “butterflies in the stomach” commonly associated with exams. However, the symptoms with the highest relative increases in prevalence were loss of appetite, frequent diarrhea and sexual indifference. These can therefore be recommended as the most target specific symptoms of exam stress for future studies. Finally, the symptoms with the strongest relative increases when counting severe/very severe ratings only, were excessive tiredness after mild exertion, back pain, nausea and headache, indicating that these are the exam stress related symptoms perceived as the most impairing. 

The differentiation of MES and MUS poses a challenging problem in the study of somatization [44]. Reports of infections and injuries were obtained in the present study to account for the effects of MES statistically. In addition these reports were found to significantly decline during exam period by about half, while predicting increased somatization symptom scores across all sessions. This decrease in MES may be explained by the well-known temporary immune-enhancing effects of acute stressors [45], as well as reductions in social and/or physical activity during the exam preparation period.

### Neuroticism is associated with somatization increases under stress

Neuroticism was found to explain a significant amount of variance in somatization under exam stress. Participants with high trait neuroticism scores showed higher symptom scores in general and higher symptom increases under exam stress. This finding replicates previous reports of baseline correlations between neuroticism and somatic symptoms [5,7,46,47] and extends them by showing that neuroticism can explain stress-induced somatization. Neuroticism has been investigated as personality trait moderating stress reactivity since a long time. It has been linked to differences in the appraisal of a stressor, as well as the reactivity to a stressor. Further it has been discussed in conjunction with differences in stress coping behaviors and even differences in exposure to stressors [48,49]. All of these influences might account for the observed relationship.

### No support for the stress-alexithymia hypothesis

Although the present study could replicate findings that TAS-alexithymia (for review see: [9]), trait anxiety and baseline depression [5] are positively associated with somatization at baseline, no such relationship could be found for increases in somatization under exam stress. Compared to neuroticism, TAS-alexithymia, anxiety and baseline depression had only a marginal likelihood of being the best explanatory variable for the observed variance in symptom index. These results do not support our second hypothesis, which claimed that alexithymia would be a significant and superior explanatory variable for exam stress induced somatization. This finding is in agreement with experimental studies, which reported that alexithymia was not associated with an increased reactivity to physiological measures of stress [12,50]. Further our results are in line with a longitudinal study, which could not find a predictive value of alexithymia for the persistence of unexplained physical symptoms in general medical outpatients [51]. In accord with earlier conclusions [52], our results point out that alexithymia and somatization might not be linked by a difference in reactivity to acute stress in the general population. It must be emphasized that exam stress is only one specific form of psychosocial stress. The link between alexithymia and stress reactivity might be different for other forms of psychosocial stress, such as long-term interpersonal stress. Moreover, the role of alexithymia might be different in patient samples, or highly alexithymic sub-populations.

### On the temporal stability of the TAS-20

The temporal stability of the TAS-20, especially in clinical samples and under conditions of psychological distress, has been a matter of discussion [37]. In the present study, significant differences in TAS-20 between session 2 and 3, but not 2 and 1 could be found. This result somewhat confirms reports of a state- and probably stress dependent proportion of TAS-20 scores [37,53]. However, the small size of session-to-session differences during a period of increased stress leads us to the conclusion that the TAS-20 is a reliable trait measure with only minor state confounds. Pearson’s correlation coefficients computed and a ICC computed for all three survey time-points confirm this notion by indicating an acceptable re-tests reliability of r>.75.

### Limitations

Two baselines, before and after exam period, were included in the present study. Since all psychometric scores returned to normal at post-exam baseline, it can be ruled out that the observed increases reflect simple time effects. A number of control variables showed parallel effects for measures of distress (stress, depression and anxiety) and a reduction in infection and/or injuries, which confirms that the observed effects are due to exam stress. MES were surveyed and categorized to control for participant bias. Nevertheless, several limitations apply to the present study and its interpretation:

The main limitation of the present study is that a self-reports bias, driven by social desirability might have confounded all measures. Participants were aware of the study’s focus on the effects of exam stress, since the retrieval of exact exam dates would otherwise not have been possible. This might have in- or deflated somatization reports during the exam period, especially since it is known that students tend to bias retrospective ratings of pre-exam distress in the direction that maximizes self-esteem [54].

In addition, our sample size decreased with each survey. This might have biased results towards a healthier student sample. Differences between pre- and post- exam baselines are therefore not discussed, since these might be explained by drifts in sample characteristics and reduced stress levels alike.

Finally, the trait measures NEO-FFI and STAI-G-X2 have been recorded only at pre- or post-baseline only, to reduce participant’s timely efforts. Despite the high temporal stability of the STAI-G-X2 [29], its comparability with the other trait variables might be confounded by subtle state-differences between the baselines and the mentioned change in sample size.

## Conclusion

Here we could show that somatization significantly increased under exam stress across a range of symptom dimensions in healthy university students. The present results verify that transient increases in somatization can be evoked by psychosocial stress. The dataset constitutes a valuable basis for future studies on bodily symptoms under psychosocial stress. Further the present findings could not support the stress-alexithymia hypothesis. This highlights that studies in search of personality factors predisposing for somatization should always consider alternative explanatory concepts. Neuroticism was identified as a better correlate of somatization induced by acute exam stress than TAS-alexithymia, trait anxiety or depression. Perceptions or behaviors related to neuroticism might be of etiological importance for somatization under psychosocial stress and pose interesting targets for future studies.

## Supporting Information

Table S1 **SOMS items significantly increasing under exam stress: Full results.** Symptoms were surveyed according to the Screening for Somatoform Symptoms 7 day (SOMS-7d). Effects of exam period were tested using Friedman’s test (Χ^2^_F_). The alpha-Level was set to ≤ 0.001 to correct for multiple comparisons. Significant results are depicted as **** p ≤ 0.0001, *** p ≤ 0.001. All post-hoc differences between baselines and exam period were significant as tested with Wilcoxon’s paired rank tests (results not shown). Increases in symptom prevalence are shown in %of valid cases for any severity (score ≥ 1) and severe/very severe only (score ≥ 3) at baseline.
(DOCX)

Supplement S1 **Additional sample information and mixed model syntax.**
(DOCX)
