# Supplementary material for: Somatic Symptoms Evoked by Exam Stress in University Students: The Role of Alexithymia, Neuroticism, Anxiety and Depression
Source: PLoS One. 2013 Dec 18;8(12):e84911. doi: 10.1371/journal.pone.0084911 (PMC3867544; doi:10.1371/journal.pone.0084911)
Supplement: Table S1 — SOMS items significantly increasing under exam stress: Full results. Symptoms were surveyed according to the Screening for Somatoform Symptoms 7 day (SOMS-7d). Effects of exam period were tested using Friedman’s test (Χ2 F). The alpha-Level was set to ≤ 0.001 to correct for multiple comparisons. Significant results are depicted as **** p ≤ 0.0001, *** p ≤ 0.001. All post-hoc differences between baselines and exam period were significant as tested with Wilcoxon’s paired rank tests (results not shown). Increases in symptom prevalence are shown in %of valid cases for any severity (score ≥ 1) and severe/very severe only (score ≥ 3) at baseline. (DOCX) [file pone.0084911.s001.docx]

**Table S 1: SOMS items significantly increasing under exam stress: Full results.**

| **Item No** | **Symptom** | **Pre-Baseline**  **n=141 (f=70, m=71)** | | | | **Exam Period**  **n=123 (f=61, m=62)** | | | | | | **Post-Baseline**  **n=117 (f=59, m=58)** | | | **Relative Increase** | | **Exam**  **Effect** | |
| --- | --- | --- | --- | --- | --- | --- | --- | --- | --- | --- | --- | --- | --- | --- | --- | --- | --- | --- |
|  |  | **Symptom index** | **%**  **Prevalence** | | | | **Symptom index** | | **%**  **Prevalence** | | **Symptom index** | | **%**  **Prevalence** | | **%**  **Baseline Prevalence** | | **Χ^2^_F_** | **Sig.** |
|  |  | Mean±SD | **Any/Severe** | | | | Mean±SD | | **Any/Severe** | | Mean±SD | | **Any/Severe** | | **Any/Severe** | |  |  |
| 1 | Headache | 0.82±0.92 | | 53.2 | 5.7 | | 1.33±1.11 | 70.7 | | 16.3 | 0.71±0.78 | | 55.1 | 3.4 | 30.7 | 258.8 | 26.70 | **** |
| 2 | Abdominal pain | 0.65±0.89 | | 42.6 | 4.3 | | 0.93±0.98 | 57.7 | | 5.7 | 0.64±0.79 | | 45.8 | 0.8 | 30.7 | 123.1 | 14.91 | *** |
| 3 | Back pain | 0.71±0.86 | | 50.4 | 2.8 | | 1.24±1.12 | 67.5 | | 15.4 | 0.75±0.84 | | 54.2 | 2.5 | 29.0 | 474.3 | 26.85 | **** |
| 4 | Joint pain | 0.33±0.69 | | 23.4 | 2.8 | | 0.59±0.88 | 37.4 | | 3.3 | 0.34±0.64 | | 25.4 | 0.8 | 53.2 | 76.5 | 14.17 | *** |
| 5 | Pain in legs/ arms | 0.29±0.54 | | 25.5 | 0.7 | | 0.51±0.80 | 36.6 | | 1.6 | 0.43±0.71 | | 33.1 | 0.8 |  |  |  |  |
| 6 | Chest pain | 0.09±0.35 | | 7.1 | 0.7 | | 0.21±0.62 | 13.8 | | 1.6 | 0.19±0.51 | | 14.4 | 0.8 |  |  |  |  |
| 7 | Rectal pain | 0.11±0.35 | | 9.2 | 0.0 | | 0.15±0.54 | 8.9 | | 1.6 | 0.06±0.33 | | 4.2 | 0.8 |  |  |  |  |
| 8 | Pain during sexual intercourse | 0.06±0.31 | | 4.3 | 0.7 | | 0.07±0.25 | 6.5 | | 0.0 | 0.08±0.32 | | 5.9 | 0.0 |  |  |  |  |
| 9 | Pain during urination | 0.06±0.34 | | 4.3 | 0.7 | | 0.08±0.35 | 6.5 | | 0.8 | 0.03±0.18 | | 3.4 | 0.0 |  |  |  |  |
| 10 | Nausea | 0.45±0.71 | | 33.3 | 0.7 | | 0.76±0.90 | 50.4 | | 3.3 | 0.41±0.66 | | 32.2 | 0.8 | 53.8 | 317.8 | 23.64 | **** |
| 11 | Bloating | 0.89±0.93 | | 57.4 | 6.4 | | 0.91±0.91 | 60.2 | | 4.9 | 0.75±0.97 | | 46.6 | 8.5 |  |  |  |  |
| 12 | Discomfort/ churning around stomach | 0.72±0.94 | | 44.0 | 6.4 | | 1.24±1.10 | 71.5 | | 14.6 | 0.54±0.83 | | 35.6 | 3.4 | 79.8 | 199.5 | 43.44 | **** |
| 13 | Vomiting (excluding pregnancy) | 0.04±0.26 | | 2.8 | 0.0 | | 0.03±0.28 | 1.6 | | 0.8 | 0.05±0.29 | | 3.4 | 0.0 |  |  |  |  |
| 14 | Regurgitation | 0.20±0.50 | | 16.3 | 0.7 | | 0.36±0.77 | 22.8 | | 3.3 | 0.11±0.31 | | 11.0 | 0.0 |  |  |  |  |
| 15 | Hiccoup or heartburn | 0.18±0.49 | | 14.9 | 0.7 | | 0.17±0.47 | 13.0 | | 0.0 | 0.17±0.42 | | 15.3 | 0.0 |  |  |  |  |
| 16 | Food intolerance | 0.23±0.58 | | 16.3 | 1.4 | | 0.33±0.76 | 19.5 | | 3.3 | 0.24±0.62 | | 16.1 | 2.5 |  |  |  |  |
| 17 | Loss of appetite | 0.30±0.68 | | 19.9 | 2.8 | | 0.67±0.97 | 41.5 | | 7.3 | 0.22±0.59 | | 15.3 | 1.7 | 136.2 | 222.9 | 26.36 | **** |
| 18 | Bad taste in mouth or coated tongue | 0.45±0.76 | | 31.9 | 3.5 | | 0.49±0.79 | 33.3 | | 3.3 | 0.32±0.64 | | 24.6 | 1.7 |  |  |  |  |
| 19 | Dry mouth | 0.24±0.57 | | 17.7 | 0.7 | | 0.38±0.75 | 26.0 | | 4.1 | 0.27±0.59 | | 20.3 | 0.8 |  |  |  |  |
| 20 | Frequent diarrhea | 0.22±0.60 | | 14.9 | 2.1 | | 0.50±0.91 | 30.1 | | 4.9 | 0.19±0.53 | | 14.4 | 0.8 | 105.3 | 227.9 | 14.24 | *** |
| 21 | Discharge of fluid from anus | 0.05±0.28 | | 3.5 | 0.0 | | 0.07±0.34 | 4.9 | | 0.0 | 0.02±0.13 | | 1.7 | 0.0 |  |  |  |  |
| 22 | Frequent urination | 0.33±0.62 | | 27.0 | 1.4 | | 0.60±0.94 | 36.6 | | 6.5 | 0.24±0.60 | | 16.9 | 1.7 |  |  |  |  |
| 23 | Frequent defecation | 0.25±0.58 | | 18.4 | 0.7 | | 0.44±0.80 | 27.6 | | 3.3 | 0.15±0.48 | | 11.0 | 0.8 |  |  |  |  |
| 24 | Palpitations | 0.13±0.42 | | 10.6 | 0.0 | | 0.33±0.70 | 23.6 | | 3.3 | 0.11±0.41 | | 8.5 | 0.8 |  |  |  |  |
| 25 | Feelings of pressure around precordium | 0.09±0.31 | | 8.5 | 0.0 | | 0.24±0.61 | 18.7 | | 1.6 | 0.13±0.44 | | 9.3 | 0.8 |  |  |  |  |
| 26 | Sweating | 0.23±0.51 | | 19.1 | 0.7 | | 0.44±0.84 | 26.8 | | 4.1 | 0.22±0.53 | | 16.9 | 0.0 |  |  |  |  |
| 27 | Flushing or blushing | 0.23±0.56 | | 17.7 | 0.7 | | 0.45±0.78 | 29.3 | | 2.4 | 0.19±0.44 | | 17.8 | 0.0 |  |  |  |  |
| 28 | Breathlessness without exertion | 0.08±0.36 | | 5.7 | 0.7 | | 0.15±0.57 | 9.8 | | 2.4 | 0.08±0.36 | | 5.9 | 0.0 |  |  |  |  |
| 29 | Hyperventilation | 0.11±0.43 | | 8.5 | 1.4 | | 0.21±0.62 | 13.8 | | 3.3 | 0.07±0.31 | | 5.1 | 0.0 |  |  |  |  |
| 30 | Excessive tiredness on mild exertion | 0.50±0.85 | | 31.9 | 5.0 | | 1.03±1.21 | 52.0 | | 17.1 | 0.37±0.62 | | 30.5 | 0.8 | 66.7 | 487.5 | 29.88 | **** |
| 31 | Blotchiness or discoloration of skin | 0.10±0.40 | | 6.4 | 0.0 | | 0.31±0.73 | 19.5 | | 2.4 | 0.09±0.39 | | 6.8 | 0.8 |  |  |  |  |
| 32 | Sexual indifference | 0.35±0.69 | | 26.2 | 2.8 | | 0.57±0.83 | 39.8 | | 4.9 | 0.23±0.55 | | 17.8 | 0.8 | 80.9 | 164.8 | 16.08 | *** |
| 33 | Unpleasant sensations around genitals | 0.11±0.39 | | 8.5 | 0.7 | | 0.14±0.50 | 9.8 | | 0.8 | 0.04±0.20 | | 4.2 | 0.0 |  |  |  |  |
| 34 | Impaired coordination or balance | 0.13±0.39 | | 10.6 | 0.0 | | 0.28±0.63 | 20.3 | | 1.6 | 0.08±0.30 | | 6.8 | 0.0 |  |  |  |  |
| 35 | Paralysis or myasthenia | 0.01±0.12 | | 1.4 | 0.0 | | 0.07±0.34 | 4.9 | | 0.0 | 0.03±0.16 | | 2.5 | 0.0 |  |  |  |  |
| 36 | Difficulty swallowing or lump in throat | 0.18±0.55 | | 11.3 | 1.4 | | 0.16±0.55 | 10.6 | | 2.4 | 0.08±0.27 | | 7.6 | 0.0 |  |  |  |  |
| 37 | Loss of voice | 0.11±0.40 | | 9.2 | 0.7 | | 0.07±0.32 | 5.7 | | 0.0 | 0.07±0.31 | | 5.1 | 0.0 |  |  |  |  |
| 38 | Urinary retention | 0.01±0.08 | | 0.7 | 0.0 | | 0.04±0.24 | 3.3 | | 0.0 | 0.03±0.21 | | 1.7 | 0.0 |  |  |  |  |
| 39 | Hallucinations | 0.06±0.23 | | 5.7 | 0.0 | | 0.10±0.32 | 8.9 | | 0.0 | 0.04±0.24 | | 3.4 | 0.0 |  |  |  |  |
| 40 | Loss of touch or pain sensation | 0.01±0.08 | | 0.7 | 0.0 | | 0.04±0.24 | 3.3 | | 0.0 | 0.04±0.20 | | 4.2 | 0.0 |  |  |  |  |
| 41 | Paresthesias | 0.14±0.41 | | 12.1 | 0.0 | | 0.25±0.64 | 17.1 | | 2.4 | 0.11±0.34 | | 10.2 | 0.0 |  |  |  |  |
| 42 | Double vision | 0.05±0.30 | | 3.5 | 0.7 | | 0.07±0.31 | 4.9 | | 0.0 | 0.01±0.09 | | 0.8 | 0.0 |  |  |  |  |
| 43 | Blindness | 0.01±0.08 | | 0.7 | 0.0 | | 0.01±0.09 | 0.8 | | 0.0 | - | | - | 0.0 |  |  |  |  |
| 44 | Deafness | 0.03±0.17 | | 2.8 | 0.0 | | 0.01±0.09 | 0.8 | | 0.0 | 0.03±0.16 | | 2.5 | 0.0 |  |  |  |  |
| 45 | Seizures | 0.01±0.12 | | 1.4 | 0.0 | | 0.07±0.34 | 4.9 | | 0.0 | 0.04±0.24 | | 3.4 | 0.0 |  |  |  |  |
| 46 | Amnesia | 0.06±0.30 | | 5.0 | 0.0 | | 0.11±0.40 | 7.3 | | 0.0 | 0.05±0.26 | | 4.2 | 0.0 |  |  |  |  |
| 47 | Fainting | 0.01±0.08 | | 0.7 | 0.0 | | - | - | | 0.0 | 0.01±0.09 | | 0.8 | 0.0 |  |  |  |  |
| 48 | Painful menstruation | 0.51±0.96 | | 27.1 | 5.7 | | 0.56±0.99 | 29.5 | | 6.6 | 0.47±0.91 | | 25.0 | 6.7 |  |  |  |  |
| 49 | Irregular menstruation | 0.41±0.96 | | 20.0 | 5.7 | | 0.67±1.22 | 31.1 | | 13.1 | 0.33±0.84 | | 18.3 | 5.0 |  |  |  |  |
| 50 | Excessive menstrual bleeding | 0.17±0.54 | | 11.4 | 1.4 | | 0.20±0.65 | 9.8 | | 3.3 | 0.15±0.49 | | 10.2 | 0.0 |  |  |  |  |
| 51 | Vomiting during pregnancy | - | | - | - | | - | - | | - | - | | - | - |  |  |  |  |
| 52 | Unusual/excessive vaginal discharge | 0.24±0.52 | | 20.0 | 0.0 | | 0.48±0.81 | 31.1 | | 3.3 | 0.31±0.65 | | 22.0 | 1.7 |  |  |  |  |
| 53 | Erectile or ejaculatory dysfunction | 0.06±0.37 | | 2.8 | 1.4 | | 0.02±0.13 | 1.6 | | 0.0 | 0.05±0.22 | | 5.2 | 0.0 |  |  |  |  |

Symptoms were surveyed according to the Screening for Somatoform Symptoms 7 day (SOMS-7d)**.** Effects of exam period were tested using Friedman’s test (Χ^2^_F_). The alpha-Level was set to ≤ 0.001 to correct for multiple comparisons. Significant results are depicted as **** p ≤ 0.0001, *** p ≤ 0.001. All post-hoc differences between baselines and exam period were significant as tested with Wilcoxon’s paired rank tests (results not shown). Increases in symptom prevalence are shown in %of valid cases for any severity (score ≥ 1) and severe/very severe only (score ≥ 3) at baseline.
