# Supplementary material for: Somatic Symptoms Evoked by Exam Stress in University Students: The Role of Alexithymia, Neuroticism, Anxiety and Depression
Source: PLoS One. 2013 Dec 18;8(12):e84911. doi: 10.1371/journal.pone.0084911 (PMC3867544; doi:10.1371/journal.pone.0084911)
Supplement: Supplement S1 — Additional sample information and mixed model syntax. (DOCX) [file pone.0084911.s002.docx]

**Supplement 1:**

**1.) Additional sample information:**

The sample size of 150 participants was selected a-priori, based on the resources available for recruiting and testing. Of the 142 participants passed to analysis, 90 participants were recruited during academic winter term 2011/2012 and 52 during summer term 2012. A mean of 126.1±48.3 days passed between study inclusion (pre-baseline) and study exclusion (post-baseline). Mean exam phase duration (last, minus first exam date) was 19.7±20.0 days, ranging from 0 to 122. Data for all variables was checked for outliers by visual examination of histograms, scatterplots and by comparison with the available reference sample distributions. No unambiguous outliers were identified.

**Excluded from study:**

Eight (6 male, 2 female) participants were excluded from the study due to the following reasons:

Acute disorder or injury (n=3); Lost to follow up (n=3); University drop out(n=1). Voluntary drop out (n=1). No reason was given for the withdrawal of consent for the voluntary drop out.

**Past/Chronic Disorders:**

Sixteen (8 male, 8 female) participants had a history of major internal or psychiatric disorders or stable chronic disorders. These were: Migraine (n=5); Chronic back pain (n=3); Chronic Joint pain (n=2); Depression (n=2); Tinnitus, Urticaria, Orofacial Pain, Social Anxiety (each n=1).

**Participants’ fields of study** (in % of valid cases.**):**

Psychology/medicine 26.8%
Humanities 27.5%
Natural sciences, engineering, mathematics or informatics 22.5%
Economics 5.6%
others in 17.6%

**Participants’ drug habits** (in % of valid cases**):**

Regular alcohol consumption: 83.7%

Regular nicotine consumption: 22.7%

Regular caffeine consumption: 66.7%

Regular consumption of cannabis/illicit drugs: 0.7%

**2.) Model Syntax**

The syntax for the mixed modeling analysis used is given below. Drop outs and sessions not fulfilling the timing criteria for survey submission have to be excluded from analysis by using the filter variable: “filter_valid_for_analysis”.

**Basic model:**

GENLINMIXED

/DATA_STRUCTURE SUBJECTS=participant REPEATED_MEASURES=time COVARIANCE_TYPE=AR1

/FIELDS TARGET=SOMS7d_intensity_index TRIALS=NONE OFFSET=NONE

/TARGET_OPTIONS DISTRIBUTION=NORMAL LINK=IDENTITY

/FIXED EFFECTS= time disorder MES USE_INTERCEPT=TRUE

/RANDOM USE_INTERCEPT=TRUE SUBJECTS= participant COVARIANCE_TYPE=DIAGONAL

/BUILD_OPTIONS TARGET_CATEGORY_ORDER=ASCENDING INPUTS_CATEGORY_ORDER=ASCENDING MAX_ITERATIONS=100 CONFIDENCE_LEVEL=95 DF_METHOD=SATTERTHWAITE COVB=ROBUST.

*** The target variable was substituted by PSQ_total, BDI_II, STAI_G_X1 and TAS_Taylor_total for analyses (aim one) and are depicted in Table 1.**

**Model with trait predictors:**

GENLINMIXED

/DATA_STRUCTURE SUBJECTS=participant REPEATED_MEASURES=time COVARIANCE_TYPE=AR1

/FIELDS TARGET=SOMS7d_intensity_index TRIALS=NONE OFFSET=NONE

/TARGET_OPTIONS DISTRIBUTION=NORMAL LINK=IDENTITY

/FIXED EFFECTS= time disorder MES NEO_FFI_Neutot_SUM_time3 time*NEO_FFI_Neutot_SUM_time3 USE_INTERCEPT=TRUE

/RANDOM EFFECTS= NEO_FFI_Neutot_SUM_time3 USE_INTERCEPT=TRUE SUBJECTS= participant COVARIANCE_TYPE=DIAGONAL

/BUILD_OPTIONS TARGET_CATEGORY_ORDER=ASCENDING INPUTS_CATEGORY_ORDER=ASCENDING MAX_ITERATIONS=100 CONFIDENCE_LEVEL=95 DF_METHOD=SATTERTHWAITE COVB=ROBUST.

*** The fixed and random effects in green were substituted by BDI_II_Mean_Baseline_all, STAI_G_X2_time1_mean_centered and TAS_Taylor_total_time3 for the analyses (aim two) and are described in the results section.**
